# Fluorescent-Based Methods for Gene Knockdown and Functional Cardiac Imaging in Zebrafish

**DOI:** 10.1007/s12033-013-9664-6

**Published:** 2013-05-15

**Authors:** Noriko Umemoto, Yuhei Nishimura, Yasuhito Shimada, Yukiko Yamanaka, Seiya Kishi, Saki Ito, Kana Okamori, Yuuki Nakamura, Junya Kuroyanagi, Zi Zhang, Liqing Zang, Zhipeng Wang, Norihiro Nishimura, Toshio Tanaka

**Affiliations:** 1 Department of Molecular and Cellular Pharmacology, Pharmacogenomics and Pharmacoinformatics, Mie University Graduate School of Medicine, 2-174 Edobashi, Tsu, Mie 514-8507 Japan; 2 Mie University Medical Zebrafish Research Center, 2-174 Edobashi, Tsu, Mie 514-8507 Japan; 3 Department of Bioinformatics, Mie University Life Science Research Center, 2-174 Edobashi, Tsu, Mie 514-8507 Japan; 4 Department of Omics Medicine, Mie University Industrial Technology Innovation Institute, 2-174 Edobashi, Tsu, Mie 514-8507 Japan; 5 Department of Translational Medical Science, Mie University Graduate School of Medicine, 2-174 Edobashi, Tsu, Mie 514-8507 Japan

**Keywords:** Zebrafish, Gene knockdown, Morpholino, Lissamine, Cardiac function, Bodipy-ceramide, Cardiac troponin T, Cardiomyopathy, Gene dosage effect, Human disease model

## Abstract

A notable advantage of zebrafish as a model organism is the ease of gene knockdown using morpholino antisense oligonucleotide (MO). However, zebrafish morphants injected with MO for a target protein often show heterogeneous phenotypes, despite controlling the injection volume of the MO solution in all embryos. We developed a method for estimating the quantity of MO injected into each living morphant, based on the co-injection of a control MO labeled with the fluorophore lissamine. By applying this method for knockdown of cardiac troponin T (*tnnt2a*) in zebrafish, we could efficiently select the partial *tnnt2a*-depleted zebrafish with a decreased heart rate and impairment of cardiac contraction. To investigate cardiac impairment of the *tnnt2a* morphant, we performed fluorescent cardiac imaging using Bodipy-ceramide. Cardiac image analysis showed moderate reduction of *tnnt2a* impaired diastolic distensibility and decreased contraction and relaxation velocities. To the best of our knowledge, this is the first report to analyze the role of *tnnt2a* in cardiac function in *tnnt2a*-depleted living animals. Our combinatorial approach can be applied for analyzing the molecular function of any protein associated with human cardiac diseases.

**Electronic supplementary material:**

The online version of this article (doi:10.1007/s12033-013-9664-6) contains supplementary material, which is available to authorized users.

## Introduction

Gene targeting technology, such as gene knockout (KO) and gene knockdown, is used in the targeted analysis of specific gene function in human disease. When downregulation, or reduced activity, of a gene product is inferred to be associated with the cause and progress of a disease, a KO animal for that gene may be developed to evaluate phenotypic similarity to the human disease. However, a particular difficulty arises when the homozygous KO results in embryonic lethality. To overcome the lethality of the homozygous KO, the phenotype of the heterozygous KO animal can be evaluated. Unfortunately, a 50 % reduction in the expression of a targeted gene in heterozygous mutants rarely results in a detectable phenotype [1, 2]. In contrast, little is known regarding phenotypes resulting from a 70 to 80 % reduction in target gene expression [1, 2]. This intermediate range may correspond to the variation associated with point mutations or reduced expression levels of alleles of human disease genes [1, 2]. For example, mutations of cardiac troponin T (*TNNT2*) are responsible for 15 % of all cases of familial hypertrophic cardiomyopathy [3]. Troponin is a complex composed of three distinct gene products, troponin C (TnC), troponin I (TnI), and troponin T (TnT) in skeletal and cardiac muscles. Homozygous *Tnnt2* KO mice embryos show severe cardiac malformation without a heartbeat and die at approximately embryonic day 10 (E10) [4, 5]. In contrast, *Tnnt2* heterozygous KO mice do not show any significant impairment in cardiac function [4, 5], making it difficult to analyze the gene dosage effect of *Tnnt2* in a beating heart using either homozygous or heterozygous KO mice. Therefore, a method is required to generate animal models exhibiting intermediate phenotypes in which expression of the gene of interest is precisely reduced [6].

Zebrafish is an attractive model organism for investigation of cardiovascular disease because of its genetic tractability, external fertilization, early optical transparency, and ability to survive without a functional cardiovascular system during development [7]. In particular, the two major advantages of zebrafish embryos are simple gene manipulation using morpholino antisense oligonucleotides (MOs) and live imaging of tissue highlighted by a fluorescent dye or expressed fluorescent proteins [8–10]. Knockdown of any target protein is readily achieved with an MO [9, 11–14]. The expression of the targeted protein is reduced by MOs in a dose-dependent manner [11, 15]. However, one of the difficulties in experiments using MOs is that the quantity of MO injected can slightly vary among embryos, reflecting the severity of the phenotype in the morphants.

To manipulate a targeted gene dosage effect, we improved this method by injecting the MO-targeted certain gene into each embryo by co-injection of control MO labeled with the fluorophore lissamine (Lis-MO). The control MO has no target in the zebrafish genome. In the current study, we determined the fluorescence intensity (FI) from Lis-MO in each morphant injected with a mixture of the targeted MO and Lis-MO, and determined the relative expression levels of mRNA for the target gene in each morphant. Although previous reports have shown that *tnnt2a* morphants with no cardiac contraction completely inhibit translation of tnnt2a protein by injecting a high concentration of *tnnt2a*-MO [16, 17], we were able to develop *tnnt2a* morphants with a homogeneous intermediate phenotype that is in between homozygous and heterozygous *Tnnt2* KO models by applying our method. In addition, to characterize ventricular impairment in the *tnnt2a* morphants with impaired cardiac function, we also performed cardiac assessment of zebrafish to measure the performance of the inner-ventricular wall using a commercially available fluorescent dye, Bodipy-ceramide [18]. In the present study, functional cardiac imaging of zebrafish stained with Bodipy-ceramide was able to detect both systolic and diastolic dysfunction in the heart, albeit a fluorescent protein-labeled heart.

## Materials and Methods

The investigation conformed to the ethical guidelines established by the Institutional Animal Care and Use Committee of Mie University.

### Zebrafish Breeding

Care and breeding of zebrafish were as described previously [19]. Poorly pigmented *nacre* mutants of zebrafish [20] were used in this study because of their greater transparency. Homozygous *nacre* mutants lack melanophores throughout development, but have increased numbers of iridophores and have a mutation in a zebrafish gene, microphthalmia-associated transcription factor a (*mitfa*). Zebrafish embryos were obtained by natural spawning of the *nacre* line.

### Injection of MOs

All MOs used in this study were purchased from Gene Tools LLC (Philomath, OR, USA) and prepared and injected as previously described [14]. As a negative control, embryos were injected with a control MO (control-MO), which theoretically has no target in the zebrafish genome. The sequence of the control-MO was 5′-GAGACTTCATCTTACCTCATATTTC-3′. We also used control-MO with lissamine attached to the 3′ end (Lis-MO) to monitor the quantity of MO for the gene of interest that was present in the embryos after microinjection. We used two MOs to knockdown *tnnt2a*. The first MO, *tnnt2a*-MO1, was used to block the splicing of exon 3 of *tnnt2a*, and to analyze the correlation between the FI of Lis-MO and the knockdown level of *tnnt2a* in the embryos. The sequence of this MO was 5′-AACATAAGACTTAACCCTCCTGCTCC-3′. The second MO (*tnnt2a*-MO2) was used to block translation of *tnnt2a* mRNA and to analyze the correlation between the FI of Lis-MO and the severity of the cardiac phenotype. The sequence of this MO was reported in a previous study [17]. We injected MOs into fertilized eggs at the 1–4-cell stage using a FemtoJet microinjector (Eppendorf, Hamburg, Germany) with constant injection pressure and injection time. The settings for the FemtoJet microinjector were 50 hPa for compensation pressure, 150 hPa for injection pressure, and 0.5 s for injection time. For knockdown of *tnnt2a*, we injected a solution of 20 μM *tnnt2a*-MO1 or 1 μM *tnnt2a*-MO2 + 50 μM Lis-MO into embryos. The negative control was 20 or 1 μM control-MO + 50 μM Lis-MO. The embryos were then allowed to develop in egg water.

### Measurement of the FI of Lissamine Conjugated to Control MO Co-injected into Zebrafish Embryos

**Fig. 1:**
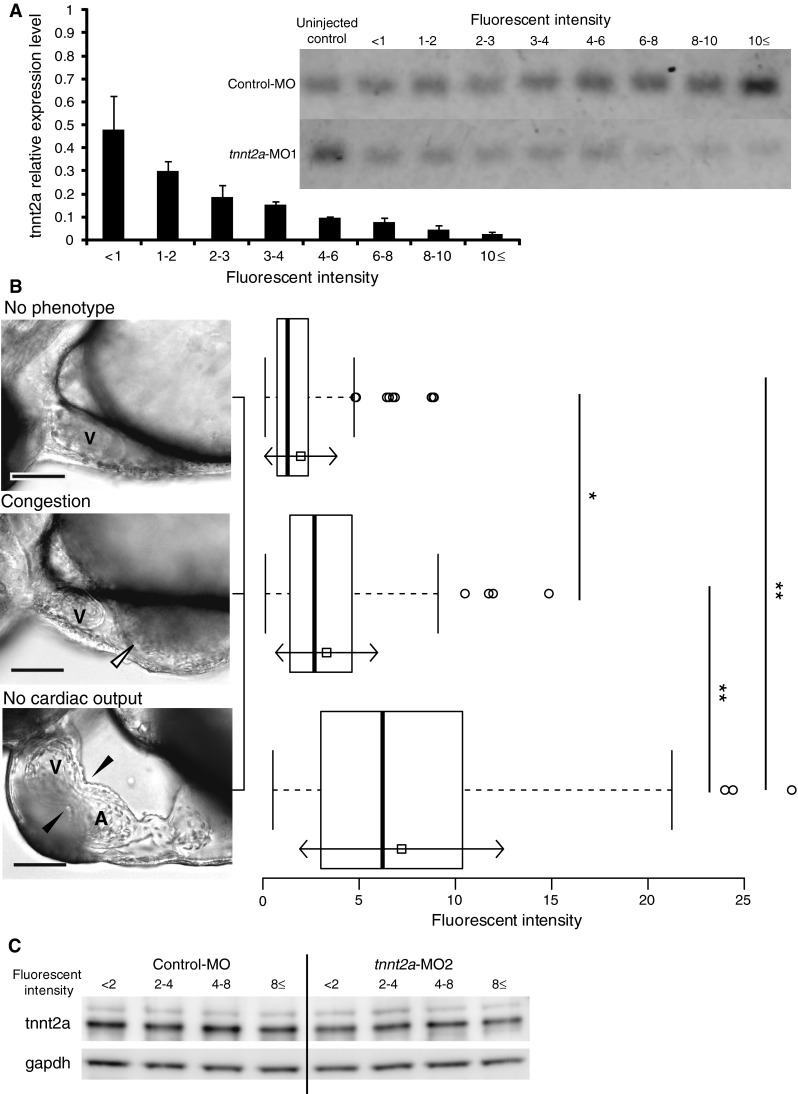
Expression level of *tnnt2a* and the cardiac phenotypes of zebrafish injected with *tnnt2a*-MO are correlated with the FI of Lis-MO in the embryo. **a** The expression level of cardiac troponin T (*tnnt2a*) was dependent on the fluorescent intensity (FI) from co-injected control antisense oligonucleotides (MO) labeled with the fluorophore lissamine (Lis-MO) in *tnnt2a* morphants classified by several ranges of the FI. **b** Zebrafish injected with 1 μM *tnnt2a*-MO2 showed three different cardiac phenotypes: no phenotype; congestion (*white arrowhead* shows accumulated blood in the sinus venosus); and no cardiac output (*black arrowhead* shows an abnormal atrioventricular valve). The FI of Lis-MO in zebrafish was significantly different among the three phenotypic groups (*p < 0.05 and **p < 0.01, Bonferroni test). These relative expressions are normalized ratios of *tnnt2a/gapdh* to the control with a FI value less than 1. The *white squares* and *arrows* in the boxplot are the mean value and ±SD of each group, respectively (no phenotype, *n* = 112; congestion, *n* = 153; no cardiac output, *n* = 183). **c** Western blotting was performed to validate the effect of *tnnt2a*-MO2. The zebrafish embryos injected with 1 μM control-MO and *tnnt2a*-MO2 were classified into four groups based on the FI from co-injected Lis-MO: (i) FI < 2, (ii) FI ≥ 2 and <4, (iii) FI ≥ 4 and <8, and (iv) FI ≥ 8. We performed an immunoblotting assay to quantify expression levels of tnnt2a protein in each group (*n* = 10–17). *Scale bar* 100 μm. *A* atrium; *V* ventricle

After anesthesia using either 0.016 % tricaine (Sigma-Aldrich, St. Louis, MO, USA) or 100 ppm 2-phenoxyethanol (Wako, Osaka, Japan), zebrafish embryos injected with MOs were placed into a 96-well plate with egg water at 60 h post-fertilization (hpf). The FI from lissamine (excitation/emission: 575/593 nm) of each zebrafish embryo was measured using a fluorescent microplate reader (Varioskan, Thermo Fisher Scientific, Waltham, MA, USA) (Fig. 1a, b).

### Quantitative PCR (qPCR) Analysis

The morphants at 60 hpf were each placed into a microcentrifuge tube and treated with RNA*later* (Ambion, Austin TX, USA) after measurement of the FI from Lis-MO. Total RNA from morphants was isolated using the RNAqueous^®^-Micro Kit (Ambion). Total RNA was used to generate cDNAs using the iScript Select cDNA Synthesis Kit (Bio-Rad, Hercules, CA, USA). Quantitative PCR for analyzing the quantity of *tnnt2a* and *gapdh* was carried out using an ABI Prism 7300 (Life Technologies, Carlsbad, CA, USA) with SYBR Green Real-time PCR Master Mix Plus (Toyobo, Osaka, Japan). The thermal cycling conditions comprised an initial step at 95 °C for 1 min, followed by 40 cycles of 95 °C for 15 s, 60 °C for 15 s, and 72 °C for 45 s. Data were normalized by the quantity of glyceraldehyde-3-phosphate dehydrogenase (*gapdh*). This allowed us to account for any variability in the initial template concentration, as well as the conversion efficiency of the reverse transcription reaction. The primers used in this study were: *tnnt2a*-F, 5′-CAACGAAGAAGTGGAAGAGTACGAG -3′; *tnnt2a*-R, 5′-TTCTCCATCGTGTTCCTGAGTG -3′; *gapdh*-F, 5′-TTCTCACAAACGAGGACACAA-3′, and *gapdh*-R, 5′-CAAGGTCAATGAATGGGTCA-3′. For visualization, the quantitative PCR product was loaded on a 2.5 % agarose gel and the gel was electrophoresed in a Mupid-2 (Cosmo Bio. Co. Ltd., Tokyo, Japan) electrophoresis tank at 50 V per cm for 80 min using 0.5× TBE buffer.

### Injection of *tnnt2a* mRNA for Phenotypic Rescue of the *tnnt2a* Morphant

The zebrafish *tnnt2a* coding region (amino acids 1–282) obtained from ZGC clone 7234566 (Open Biosystems, Huntsville, AL, USA) was subcloned into the *Eco*RI and *Xba*I sites of the plasmid pCS2P+ (Addgene, Cambridge, MA, USA). The sequence of the construct was confirmed by automated DNA sequencing. The sequence containing full-length cDNA for *tnnt2a* was amplified by PCR using the following primer pairs: 5′-TGAGATACCTACAGCGTGAGC-3′ and 5′-CCGAGATAGGGTTGAGTGTTG-3′. The PCR product was used as the template for RNA synthesis. Messenger RNA for *tnnt2a* was synthesized using the mMessage mMachine transcription kit for SP6 RNA polymerase (Ambion). For the rescue experiment, a solution containing 0.13 ng/nl of *tnnt2a* mRNA (approximately 0.39 μM) and 1 μM of *tnnt2a*-MO2 was injected into 1–4-cell stage embryos, which were then raised in egg water at 28 °C until 60 hpf.

### Protein Isolation and Western Blot Analysis

Embryos injected with MO were dechorionated at 60 hpf and were lysed in Tissue Protein Extraction Reagent (T-PER, Thermo Fisher Scientific) containing protease inhibitor cocktail (Thermo Fisher Scientific). The Pierce^®^ BCA™ protein assay kit (Thermo Fisher Scientific) was used to determine protein concentrations. The protein extract (25 μg) was loaded onto 4–12 % sodium dodecyl sulfate polyacrylamide gels, electrophoresed, and transferred to polyvinylidene fluoride (PVDF) membranes using NuPAGE and iBlot Systems (Invitrogen, Carlsbad, CA, USA). After electroblotting, membranes were blocked in PVDF Blocking Reagent for Can Get Signal^®^ (Toyobo) at room temperature or 37 °C for 1 h and probed with primary antibodies at 4 °C overnight as follows: Ab-1 (Thermo Fisher Scientific) against tnnt2a using a 500× dilution in Can Get Signal^®^ solution 1 (Toyobo); and ab36840 (Abcam, Cambridge, MA, USA) against gapdh, using a 3000× dilution in Can Get Signal^®^ solution 1. The membranes were washed thrice in phosphate-buffered saline (PBS) supplemented with 0.05 % Tween 20 (PBS-T) and incubated with horseradish peroxidase-conjugated secondary antibody (Abcam) diluted in Can Get Signal^®^ solution 2 for 1 h at room temperature. The membranes were washed again thrice in PBS-T and developed with ImmunoStar^®^ LD (Wako).

### Fluorescent Cardiac Imaging of Zebrafish Using Bodipy-ceramide

Zebrafish embryos were dechorionated and immersed for 3–5 h in egg water containing 0.2 μM of Bodipy-ceramide (BODIPY^®^ FL C5-ceramide, Invitrogen). The stained embryos were placed on a coverslip and embedded in 3 % methylcellulose solution. Embryos were arranged with the anterior to the left of the field and ventral surface down. In this position, the ventricle was clearly visible using an inverted florescence microscope (Axiovert 200M, Carl Zeiss, Oberkochen, Germany). Finally, the embryo was covered with 2 % ultralow gelling agarose (Agarose Type VII, A4018, Sigma-Aldrich) to fix its position. A coverslip was placed on the stage of the microscope (Thermo plate, Tokai Hit, Shizuoka, Japan) and maintained at 28 °C. Time-lapse images of the zebrafish ventricle were recorded at approximately 20 frames per second using AxioVision software (Rel. 4.6, Carl Zeiss) and a digital camera (AxioCam, Carl Zeiss). The imaging duration was 5 s using a GFP filter and 15 s for bright field.

### Cardiac Image Analysis Using Bodipy-ceramide Staining

**Fig. 2:**
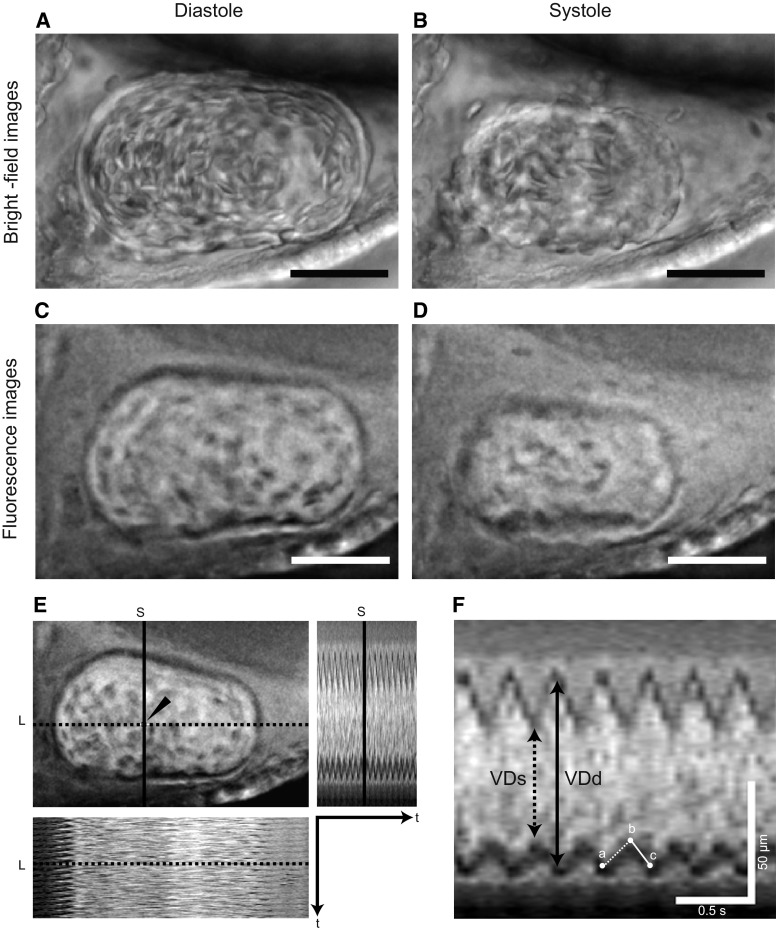
Fluorescent cardiac imaging of zebrafish ventricles using Bodipy-ceramide. **a**–**d** The images display representative individual time-lapse images from bright-field and Bodipy-ceramide stained embryos. In fluorescence images at ventricular diastole (**c**) and systole (**d**), the boundaries between the cardiac wall and the lumen can be more clearly distinguished than in bright-field images at ventricular diastole (**a**) and systole (**b**). *Scale bar* 50 μm. **e** To generate M-mode images, the intersection of the *horizontal* (*long*) axis and the *vertical* (*short*) axis lines of the “orthogonal views” was positioned at the “center of mass” (*arrowhead*). M-mode images were then generated, with each slice representing the “pseudo-linescan” of a single-pixel-wide line along these horizontal and vertical lines. M-mode images were created from serial time-lapse images of the moving ventricle with Bodipy-ceramide staining. *L* long axis, *S* short axis, *t* time. **f** The mean ventricular wall velocities during systole (mVWVs) or diastole (mVWVd) were calculated as the *a*–*b* slope and *b*–*c* slope, respectively

We used MBF ImageJ (NIH, Bethesda, MD, USA) [21] to process the original records for the measurement of cardiac performance using Bodipy-ceramide cardiac imaging. The “iterative deconvolution” plugin was used to enhance the contrast between the plasma and the ventricular wall. The inner perimeter of the ventricle in the end systolic frame was outlined using a mouse or graphic tablet, from which its “center of mass”, and subsequently, the best-fitting ellipse, were calculated [22]. The coordinates of the center of mass were memorized using the region of interest manager tool in MBF ImageJ. To make M-mode images in the long or short axes of the ventricle, the intersection of horizontal and vertical lines of the “orthogonal view” was positioned at the center of mass. M-mode images were then generated, with each slice representing a “pseudo-linescan” a single-pixel-wide along these horizontal and vertical lines. The ventricular end systolic dimension (VDs) and the ventricular end diastolic dimension (VDd) were measured from the M-mode images. VDs and VDd were obtained at maximal inward and outward excursions, respectively, of the ventricular wall in the M-mode images. We measured VDs and VDd along the short axis (short VDs, short VDd) and the long axis (long VDs, long VDd). For each determination, five diastoles and systoles were analyzed. The end diastolic volume (EDV) and end systolic volume (ESV) were calculated using the formula for a prolate spheroid (4 × *a* × *b* ^2^/3) [22]. The stroke volume (SV) was obtained by subtracting the ESV from the EDV. The ejection fraction (EF) was calculated as SV/EDV and the percentage fractional shortening (%FS) was calculated from the formula: 100(short VDd − short VDs)/short VDd. We also obtained coordinate data from a total of 11 points, representing the maximal inward (6 points) and outward (5 points) excursions of the ventral ventricular wall in the M-mode images. Mean ventricular wall velocities during systole (mVWVs) or diastole (mVWVd) were averages of five slopes at systole or diastole, respectively (Fig. 2f). We also counted the number of heartbeats for 15 s, as described previously [13].

### The Time–Volume Curve (TVC) and Its Differentiation Curve

The inner perimeter of the ventricle in each frame was outlined using the freehand selection tool in MBF image J. The lengths of the long and short axes of the ventricle in each frame were calculated from the perimeter of each ventricle using the best-fitting ellipse algorithm. Therefore, the ventricular volumes in each frame were calculated based on these axes data and entered into a volume curve differentiation program (VCDiff, Fujifilm RI Pharma, Tokyo, Japan) [23]. In this program, Fourier curve fitting was performed using five harmonics, and cardiac parameters were automatically calculated from the TVC and its differentiation curve (Fig. 4a) as follows: EDV (pl) = V1; ESV (pl) = V2; EF (%) = 100(V1 − V2)/V1; first third EF (1/3EF,  %) = 100(V1 − V3)/V1; first third filling fraction (1/3FF,  %) = 100(V4 − V2)/(V1 − V2); time to peak ejection (TPE, ms) = TD1; time to peak filling (TPF, ms) = TD2; first third ejection rate (1/3ER, pl/ms) = −D3 × 1,000/V1; peak ejection rate (PER, pl/ms) = −D1 × 1,000/V1; first third filling rate (1/3FR, pl/ms) = D4 × 1,000/T × V1; and peak filling rate (PFR, pl/ms) = D2 × 1,000/V1.

### Statistical Analysis

Statistical analysis was performed using SAS version 9.1 (SAS Institute, Cary, NC, USA) and R version 2.15.1 [24]. Spearman’s correlation coefficient by rank test was used to analyze the correlation between the expression level of *tnnt2a* and the FI from lissamine in each *tnnt2a* morphant. Significant differences for the mean FIs and cardiac parameters among the three groups were identified using the Bonferroni multiple comparisons test. Determination of the statistical difference for cardiac parameters was determined using the two-sided Student’s *t* test for comparison of two groups. The Chi square test was used to analyze the relationship between cardiac phenotype and the level of *tnnt2a* mRNA. Differences with *p* < 0.05 were considered significant.

## Results

### The Knockdown Level of *tnnt2a* in Zebrafish Injected with *tnnt2a*-MO Can be Estimated by the FI of Co-injected Lissamine-MO

To estimate the amount of MO present in each embryo after microinjection, a mixture of an MO targeted to the gene of interest and Lis-MO was injected into embryos at the 1–4-cell stage. We performed co-injection of Lis-MO to measure the knockdown efficiency of *tnnt2a* in zebrafish embryos. We designed *tnnt2a*-MO1 to knockdown *tnnt2a* by skipping exon 3 and quantified the expression level of *tnnt2a* in the morphants classified by several ranges of FI level by qPCR analysis. The FI from lissamine was assumed to co-vary with the quantity of MO injected into the eggs. As shown in Fig. 1a, the expression level of *tnnt2a* was dependent on the FI from co-injected Lis-MO in *tnnt2a* morphants (Fig. 1a). This observation suggested that the FI from Lis-MO co-injected with any MO-targeted gene of interest could be used to indirectly estimate the knockdown efficiency of the gene.

### The Severity of Cardiac Impairment in *tnnt2a* Morphants is Dependent on the FI of Co-injected Lissamine-MO

We injected a mixture of *tnnt2a*-MO2 to knockdown *tnnt2a* by blocking translation and Lis-MO at 50 μM. The *tnnt2a* morphants showed three different phenotypes at 60 hpf: (i) no phenotype; (ii) congestion; and (iii) no cardiac output (Fig. 1b). To analyze the correlation between the severity of cardiac impairment and the FI from co-injected Lis-MO, we measured the FI from Lis-MO and classified the morphants into these cardiac phenotypes (Fig. 1b, boxplots). The Bonferroni multiple comparisons test showed that the mean FI values were significantly different among the three cardiac phenotypes (no phenotype vs. congestion, *p* < 0.05; no phenotype vs. no cardiac output and congestion vs. no cardiac output, *p* < 0.01). The severity of cardiac impairment in the morphants injected with *tnnt2a*-MO1, which was mentioned above, was also dependent on the FI from co-injected Lis-MO (Fig. S1). Representative movies of the morphant and the control are shown in Supplemental movies 1–6. The severity of cardiac impairment was dependent on the FI, which was also correlated with the expression levels of *tnnt2a* in the morphants. Therefore, we conclude that co-injection of Lis-MO permits the selection of morphants with expression levels reduced by MO.

### The Cardiac Phenotypes of Zebrafish Injected with *tnnt2a*-MO are Attributed to Knockdown of tnnt2a Protein

To confirm whether the cardiac phenotypes in zebrafish injected with *tnnt2a*-MO2 were caused by knockdown of *tnnt2a*, we quantified the level of tnnt2a protein expression in zebrafish embryos using immunoblotting. As shown in Fig. S2A, the major band corresponding to a splicing variant of *tnnt2a* [25] was reduced in zebrafish injected with *tnnt2a*-MO2 at 10 μM. Morphants injected with *tnnt2a*-MO2 at 10 μM showed a weak or non-beating heart with severe abnormal morphology, which was correlated with the FI from co-injected Lis-MO (Fig. S2B-M). Representative movies of the morphant and the control are shown in Supplemental movies 7–12. Subsequently, we also detected a decrease in tnnt2a protein in the tnnt2a morphants, which showed a FI greater than 8.0, when injected with *tnnt2a*-MO2 at 1 μM. Moreover, we observed a slight reduction in the expression level of tnnt2a protein in morphants with an FI up to 8.0 from Lis-MO. This finding is probably because of the insufficient sensitivity of immunoblotting to accurately quantify tnnt2a protein levels in zebrafish (Fig. 1C, Fig. S3).

To confirm the specificity of the knockdown effect of *tnnt2a*-MO2 on the cardiac phenotypes, we co-injected *tnnt2a* mRNA with *tnnt2a*-MO2 into zebrafish eggs. At 60 hpf, 13 of 15 zebrafish embryos injected with *tnnt2a*-MO2 without *tnnt2a* mRNA showed congestion in the sinus venosus (Table 1). In contrast, only four of 20 zebrafish embryos injected with *tnnt2a*-MO2 with *tnnt2a* mRNA showed congestion (Table 1). Cardiac impairment was significantly improved by co-injection of *tnnt2a* mRNA (*p* < 0.05, Chi square test), which suggested that the cardiac phenotype induced by injection of *tnnt2a*-MO2 could be attributed to the knockdown of *tnnt2a*.

**Table 1:** Phenotypic rescue of *tnnt2a* morphants by co-injection of *tnnt2a* mRNA

	Cardiac phenotypes		Total
	No phenotype	Congestion	
tnnt2a-MO2	2	13	15
tnnt2a-MO2 + tnnt2a mRNA	16	4	20
Total	18	17	35

### Development of Fluorescent Cardiac Image Analysis Using Bodipy-ceramide Staining to Assess Cardiac Performance in Zebrafish

To analyze the cardiac function of *tnnt2a* in zebrafish with respect to cardiac output, it is important to establish methods to measure the internal ventricular dimensions. For this purpose, the boundary between the ventricular wall and the lumen must be clearly defined. However, it is difficult to detect the boundary in bright-field images because the cardiac wall of zebrafish embryos is transparent (Fig. 2a, b). To visualize the cardiac lumen, we used Bodipy-ceramide, which is used to visualize blood flow in the zebrafish heart for the analysis of the intracardiac fluid force [26] and to detect abnormal valvulogenesis during heart development [27]. Zebrafish embryos stained with Bodipy-ceramide showed a clear boundary between the ventricular wall and the lumen (Fig. 2c, d). After Bodipy-ceramide staining, the beating ventricle was recorded using fluorescence microscopy. M-mode images were created from fluorescence time-lapse images of the ventricle (Fig. 2e), and from these images, we measured cardiac parameters, including VDs, VDd, mVWVs, and mVWVd (Fig. 2f). Our data showed that fluorescent cardiac imaging with Bodipy-ceramide could be used to assess cardiac function in zebrafish.

The reliability of cardiac imaging using Bodipy-ceramide for measurement of cardiac function was tested on the developing heart of zebrafish. The sequence of cardiac development is as follows (Fig. S4A): heart tube formation at 22 hpf, looping at 36 hpf, valve formation at 48 hpf, and adult configuration at 120 hpf [28–30]. We assessed cardiac function at 60, 72, and 96 hpf because, at these stages, zebrafish have been reported to exhibit marked changes in cardiac performance [31]. In our study, mean VDs and VDd were decreased along with both short and long axes between 60 and 72 hpf (*p* < 0.05, Fig. S4B), and between 60 and 96 hpf (*p* < 0.05, Fig. S4B). Consequently, ventricular volumes (ESV, EDV) and SV were significantly lower at 72 and 96 hpf than those at 60 hpf (*p* < 0.05, Fig. S4C), while EF and %FS were increased from 60 to 96 hpf (*p* < 0.05, Fig. S4D). The 72 and 96 hpf values for cardiac parameters (ventricular dimension, ventricular volume, EF and %FS) were all significantly different from the 60 hpf values, except for the long VDs and EF values at 72 hpf (Fig. S4B–E, *p* < 0.05, Bonferroni tests). Contraction velocity, mVWVs, was significantly higher at 72 and 96 hpf compared with that at 60 hpf, and corresponding (non-significant) changes were observed in mean mVWVd (*p* < 0.05, Fig. S4E).

### Knockdown of *tnnt2a* Impairs Diastolic Distensibility and Cardiac Wall Velocity

**Fig. 3:**
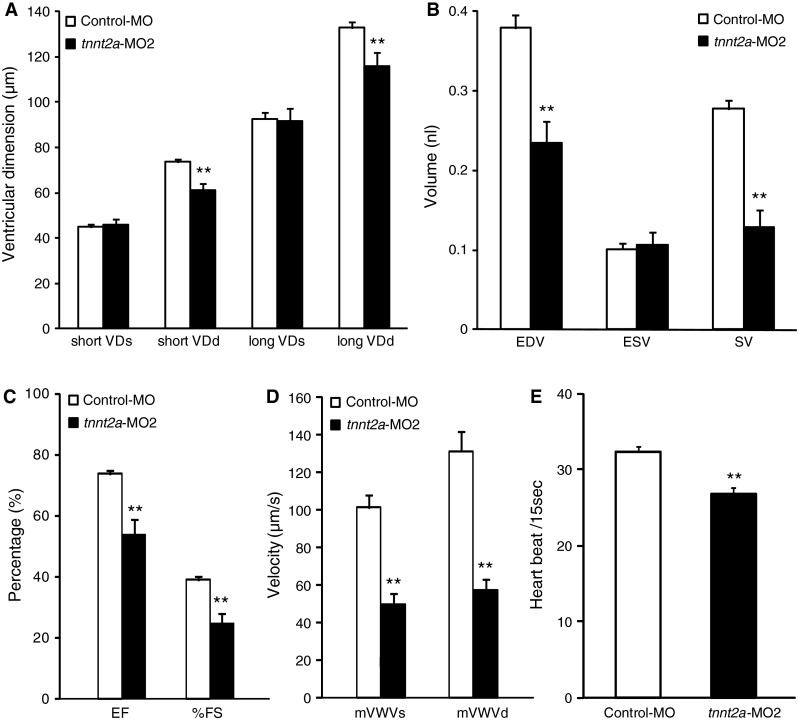
Impairment of diastolic distensibility and ventricular wall velocities during systole and diastole in zebrafish by knockdown of *tnnt2a.* In the control group, zebrafish were injected with control MO (1 μM) and Lis-MO (50 μM). In the *tnnt2a* knockdown group, zebrafish were injected with *tnnt2a*-MO2 (1 μM) and Lis-MO (50 μM). We selected zebrafish embryos, which showed FI values between 2 and 5 in the control group at 60 h post-fertilization (hpf), and compared them with *tnnt2a* morphants with blood congestion and FI values from 2 to 5 in the *tnnt2a* knockdown group at 60 hpf. **a**–**d** Cardiac parameters were measured from M-mode images of ventricles in groups of zebrafish injected with either control-MO or *tnnt2a*-MO2 at 60 hpf: **a** The ventricular end systolic dimension (VDs) and the ventricular end diastolic dimension (VDd) of both short and long axes; **b** the end diastolic volume (EDV) and end systolic volume (ESV) and stroke volume (SV); **c** ejection fraction (EF) and fractional shortening (%FS); and **d** mVWVs and mVWVd. Values are mean ± SE (control-MO, *n* = 24; *tnnt2a*-MO2, *n* = 10). Statistical analysis was performed to examine significant differences from the mean control values (**p < 0.01, *p < 0.05, Student’s *t* test)

We focused on *tnnt2a* morphants with congestion in the sinus venosus for further analysis of cardiac function because congestion might reflect a circulatory abnormality induced by reducing *tnnt2a* expression. We chose *tnnt2a* morphants with FI values between 2 and 5 because the percentage of *tnnt2a* morphants with congestion in the sinus venosus was highest in this group. Fluorescent cardiac image analysis was applied to this group. We found that, in zebrafish injected with *tnnt2a*-MO2, VDd, but not VDs, was significantly reduced compared with that in control-MO injections (*p* < 0.01, Fig. 3a). Accordingly, EDV and SV, but not ESV, were significantly reduced compared with control values (*p* < 0.01, Fig. 3b). EF and %FS were unchanged in zebrafish injected with *tnnt2a*-MO2 (Fig. 3c). These results suggested that knockdown of *tnnt2a* expression selectively impaired diastolic distensibility, which led to congestion in the sinus venosus. From the M-mode images, we also measured ventricular wall velocities during systole and diastole. Both mVWVs and mVWVd were significantly reduced in zebrafish injected with *tnnt2a*-MO2 compared with those in controls (*p* < 0.01, Fig. 3d). These results suggested that the knockdown of *tnnt2a* expression delayed ventricular wall motion equally during contraction and relaxation.

**Fig. 4:**
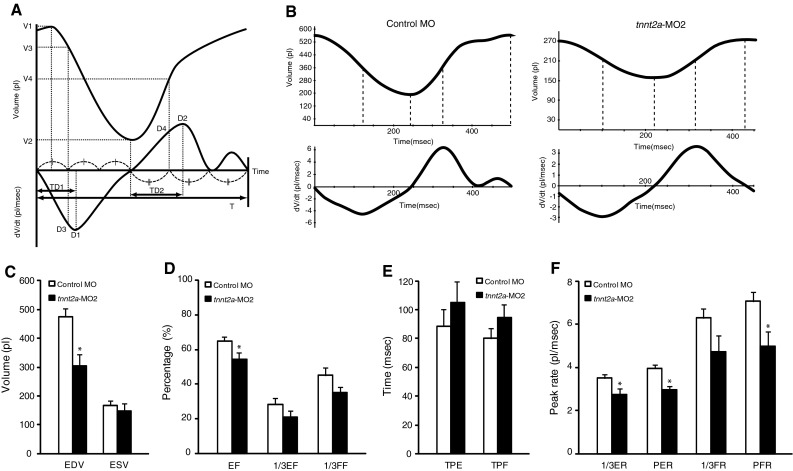
Time–volume curve (TVC) analysis showing decreased EDV, EF, and peak rates during systole and diastole by knockdown of *tnnt2a.* We also assessed cardiac parameters of both experimental groups using TVC analysis. **a** The *upper* and *lower graphs* show the TVC and the differentiation curve, respectively. **b** The TVC and the differentiation curve of control zebrafish are shown (*left*) alongside those of a *tnnt2a* morphant with congestion in the sinus venosus (*right*). **c**–**f** Cardiac function was assessed using TVC analysis based on fluorescent cardiac images using Bodipy-ceramide. The calculated parameters were as follows: **c** EDV and ESV; **d** EF, first third EF (1/3EF), and first third filling fraction (1/3FF); **e** time to peak ejection (TPE) and time to peak filling (TPF); and **f** first third ejection rate (1/3ER), peak ejection rate (PER), first third filling rate (1/3FR) and peak filling rate (PFR). Values are mean ± SE (control MO, *n* = 24; *tnnt2a*-MO2, *n* = 10). Statistical analysis was performed to examine significant differences from the mean control values (*p < 0.05, Student’s *t* test)

In addition, we performed TVC analysis using fluorescent cardiac images (Fig. 4a, b). In zebrafish injected with *tnnt2a*-MO2, EDV and EF were significantly decreased compared with those in controls (*p* < 0.05, Fig. 4c, d), consistent with observations using the M-mode method. PER and PFR were significantly decreased in zebrafish injected with *tnnt2a*-MO2 compared with those in zebrafish injected with control-MO (Fig. 4f, *p* < 0.05), but TPE and TPF were not significantly different between the groups (Fig. 4e). Ventricular wall velocities (mVWVs and mVWVd) and peak rates (PER and PFR) during systole and diastole showed velocities of contraction and relaxation that were reflected by systolic function and diastolic function, respectively. These results suggested that velocities of contraction and relaxation were lower in *tnnt2a* morphant ventricles than those in control zebrafish. Consistent with these findings, the heart rate of *tnnt2a* morphants was significantly decreased compared with that in control zebrafish (*p* < 0.01, Fig. 3e).

## Discussion

### Co-injection of a Fluorophore-Labeled MO with the MO for the Gene of Interest is Useful for Classifying Phenotypes Based on the Knockdown Level of the Gene

In practice, the quantity of MO actually injected into embryos can vary to some extent, even when they are injected with nominally similar quantities of MO. The embryos show multiple phenotypes, because the knockdown efficiency of MO depends on the quantity of MO in each morphant [15]. Therefore, it is important to measure the knockdown efficiency of the target protein in the morphants.

To evaluate the knockdown efficiency of MOs, Kamachi et al. [15] generated a modified luciferase assay, which involved two steps: (i) synthesis of the RNA containing 5′-UTR and a short N-terminal coding sequence of the target gene fused with mRNA coding luciferase; and (ii) co-injection of MO for the target and the fused RNA. However, this method is unable to evaluate the knockdown efficiency in living embryos. It is also complex to prepare the fused RNA for each target protein.

In an alternative approach, in the current study, we indirectly estimated the knockdown efficiency in living embryos co-injected with Lis-MO and a MO to knockdown the target protein, and then measured the FI of the Lis-MO in each morphant. When we validated this method with the MO to knockdown *tnnt2a*, the expression levels of *tnnt2a* mRNA in the morphants were negatively correlated with their FI. Therefore, our method has the advantage of being able to generate gene knockdown models showing relatively mild phenotypes caused by reduction of expression of a particular gene. However, a disadvantage of our method is that the knockdown effect of MO gradually disappears over approximately 7 days post-fertilization (dpf). Because the number of nuclei greatly increases during development of zebrafish embryos, the intranuclear concentration of MOs may significantly decrease, thereby reducing their knockdown effect [14]. However, our new method is useful for analysis of the effect of expression levels of a gene-of-interest on biological function, at least during developmental stages. These results confirm that measurement of FI of Lis-MO in living embryos is a convenient and reliable assay to estimate knockdown levels of a target protein.

### Cardiac Function of Zebrafish Can be Quantitatively Assessed by Fluorescent Cardiac Imaging Using Bodipy-ceramide

Zebrafish are a valuable animal model for cardiovascular research because their high transparency allows their beating hearts to be easily observed under the optical microscope. However, measurements of cardiac parameters in zebrafish are subject to inaccuracy because of difficulties in the identification of the boundary between the cardiac wall and the lumen of the heart. In previous reports, transgenic zebrafish were generated that enabled the cardiac wall to be visualized based on the expression of fluorescent proteins in the wall (e.g., Tg(cmlc2::GFP) and SAG4A) [32–34]. Because not all laboratories have access to these transgenic zebrafish, a novel method to assess cardiac function without these fish lines will be useful for cardiovascular research by using zebrafish as a model animal. In the current study, we applied fluorescent imaging technology using commercially available Bodipy-ceramide for assessment of cardiac function in zebrafish. Previously, Bodipy-ceramide has been used to visualize blood flow for the analysis of the intracardiac fluid force [26] and to detect abnormal valvulogenesis [27]. In particular, Hove et al. [26] used Bodipy-ceramide to visualize the blood flow pattern inside the heart chambers by labeling blood serum for high-speed fluorescent confocal imaging. Vermot et al. [27] also used Bodipy-ceramide to visualize valve formation and blood flow patterns using high-speed fluorescent confocal imaging. In our study, we showed that fluorescent cardiac imaging with Bodipy-ceramide enhanced the contrast of the boundary between the cardiac wall and the lumen, permitting measurement of many functional parameters, including end systolic dimension, end diastolic dimension, ESV, EDV, EF, %FS, mVWVs, and mVWVd from M-mode images, and other parameters from TVCs (Fig. 4a). Using fluorescent cardiac imaging, we found that intraventricular volume significantly decreased from 60 to 96 hpf during development of the heart. The reduction in SV and the increase in mVWVs at these developmental stages are consistent with previous reports [22, 30, 31]. Additionally, cardiac trabeculae start to form in the ventricle at approximately 72 hpf, and the ventricle becomes extensively trabeculated by 5 dpf [35]. Cardiac trabeculation might be responsible for reduction of ventricular volume from 60 to 96 hpf. These results suggested that cardiac imaging with Bodipy-ceramide reliably measured cardiac function in zebrafish.

### Characterization of Cardiac Function in the *tnnt2a* Morphant Using Fluorescent-Based Quantitative Assays

By using the two methods developed in this study, we were able to analyze cardiac function of *tnnt2a* morphants showing congestion in the sinus venosus. To the best of our knowledge, this study is the first report to reveal cardiac dysfunction induced by depletion of *tnnt2a* using a vertebrate with a beating heart.

We found that VDd, but not VDs, of *tnnt2a* morphants was significantly shorter than that in control zebrafish. TnT consists of an extended amino-terminal portion, which lies alongside tropomyosin (Tm) on the thin filament, and a globular carboxy-terminal domain that binds to Tm, as well as to TnI and TnC. TnT is involved in distributing the inhibitory effect of the troponin complex, via Tm, to seven actin monomers with which it interacts, in the absence Ca^2+^. TnT is also involved in removing this inhibitory effect from all seven actin monomers, as well as activating the actomyosin ATPase, in the presence of Ca^2+^ [36]. Many mutations related to cardiomyopathy have been reported in *TNNT2* [3, 36, 37]. In transgenic mice with *Tnnt2*-R92Q, which show weaker binding to Tm than in wild-type *Tnnt2*, left ventricular end diastolic volume, but not left ventricular end systolic volume, is significantly reduced compared with that in control mice. Conversely, in transgenic mice with *Tnnt2*-R141W, which show stronger binding to Tm than in wild-type *Tnnt2*, left ventricular end-diastolic volume is significantly increased compared with that in control mice [38]. These results suggest that impairment of diastolic distensibility in the *tnnt2a* morphant may be caused by decreased binding to Tm.

Our analysis also showed that the ventricular wall velocity for contraction (mVWVs and PER) and relaxation (mVWVd and PFR) were significantly decreased in *tnnt2a* morphants compared with those of control zebrafish. Interestingly, both maximum shortening and re-lengthening velocity of isolated ventricular cardiomyocytes in *Tnnt2*-R92Q transgenic mice are significantly decreased, which is also consistent with our observations [39]. These results suggest that there may be a common pathophysiological mechanism between knockdown of *tnnt2a* and *Tnnt2*-R92Q. Further studies are required to examine this possibility.

## Conclusions

Our combinatorial approach developed in this study can be applied for analyzing molecular function of proteins associated with human cardiac diseases, especially when the homozygous KO is lethal and the heterozygous KO animal shows no significant abnormality.

## Electronic supplementary material

Below is the link to the electronic supplementary material.

Fig. S1. Severity of cardiac impairment in the morphants injected with the *tnnt2a*-MO blocking splice site is correlated with the FI from co-injected Lis-MO. We showed that morphants injected with *tnnt2a*-MO1 (blocking splice site of *tnnt2a*) showed severe cardiac phenotypes. (A–F) Zebrafish injected with 20 μM control-MO did not show significant abnormalities in their beating heart. These images were captured under a Cy3 filter (A–C) and bright field (D–F) of an inverted florescence microscope focused on the whole body and pericardial area, respectively. (G–L) However, zebrafish injected with 20 μM *tnnt2a*-MO1 showed a weak (J and K) or non-beating heart (L) with severely abnormal morphology, which was correlated with the FI from co-injected Lis-MO. These images were captured under a Cy3 filter (G–I) and bright field (J–L) of an inverted florescence microscope focused on the whole body and pericardial area, respectively (EPS 8892 kb)

Fig. S2. Severity of cardiac impairment in the morphants injected with high dose *tnnt2a*-MO blocking translation is correlated with the FI from co-injected Lis-MO. (A) Western blotting was performed to validate the effect of *tnnt2a*-MO2. (B–G) Zebrafish injected with 10 μM control-MO did not show significant abnormalities in their beating heart. These images were captured under a Cy3 filter (B–D) and bright field (E–G) of an inverted florescence microscope focused on the whole body and pericardial area, respectively. (H–M) However, we observed that the morphants injected with high dose *tnnt2a*-MO2 (blocking translation of *tnnt2a*) showed severe cardiac phenotypes. Zebrafish injected with 10 μM *tnnt2a*-MO2 showed a weak (K) or non-beating heart (L and M) with severely abnormal morphology, which was correlated the FI from co-injected Lis-MO. These images were captured under a Cy3 filter (H–J) and bright field (K–M) of an inverted florescence microscope focused on the whole body and pericardial area, respectively (EPS 13261 kb)

Fig. S3. Densitometric immunoblotting analysis to quantify expression levels of tnnt2a protein in morphants injected with *tnnt2a*-MO2. We performed immunoblotting with densitometric analysis of Fig. 1C to quantify expression levels of tnnt2a protein in morphants injected with *tnnt2a*-MO2. Expression levels of tnnt2a protein in the tnnt2a morphant were slightly reduced (EPS 836 kb)

Fig. S4. Assessment of cardiac function in zebrafish during cardiac development by fluorescent cardiac image analysis using Bodipy-ceramide. (A) The time course of cardiac development in zebrafish. Fluorescent cardiac imaging using Bodipy-ceramide was performed using zebrafish embryos at 60, 72, and 96 hpf. (B–E) Cardiac function was assessed using M-mode images generated from the cardiac images using Bodipy-ceramide. The calculated parameters were: (B) VDs and VDd of both short and long axes; (C) EDV, ESV, and SV; (D) EF and %FS; and (E) mVWVs and mVWVd. Values are mean ± SE (60 hpf, n = 24; 72 hpf, n = 21; 96 hpf, n = 19). Statistical analysis was performed to examine significant differences among the three groups (*p < 0.05, Bonferroni test). (EPS 1168 kb)

Supplemental movie S1. Representative movies of the heart in zebrafish injected with 20 μM control-MO at 60 hpf as shown in Fig. S1D (AVI 4284 kb)

Supplemental movie S2. Representative movies of the heart in zebrafish injected with 20 μM control-MO at 60 hpf as shown in Fig. S1E (AVI 5319 kb)

Supplemental movie S3. Representative movies of the heart in zebrafish injected with 20 μM control-MO at 60 hpf as shown in Fig. S1F (AVI 4113 kb)

Supplemental movie S4. Representative movies of the heart in zebrafish injected with 20 μM *tnnt2a*-MO1 at 60 hpf. Movie S4: the morphant shows low FI (Fig. S1J) (AVI 4704 kb)

Supplemental movie S5. Representative movies of the heart in zebrafish injected with 20 μM *tnnt2a*-MO1 at 60 hpf. Movie S5: the morphant shows intermediate FI (Fig. S1K) (AVI 5542 kb)

Supplemental movie S6. Representative movies of the heart in zebrafish injected with 20 μM *tnnt2a*-MO1 at 60 hpf. Movie S6: the morphant shows high FI (Fig. S1L) (AVI 4463 kb)

Supplemental movie S7. Representative movies of the heart in zebrafish injected with 10 μM control-MO at 60 hpf as shown in Fig. S2E (AVI 6079 kb)

Supplemental movie S8. Representative movies of the heart in zebrafish injected with 10 μM control-MO at 60 hpf as shown in Fig. S2F (AVI 5909 kb)

Supplemental movie S9. Representative movies of the heart in zebrafish injected with 10 μM control-MO at 60 hpf as shown in Fig. S2G (AVI 4479 kb)

Supplemental movie S10. Representative movies of the heart in zebrafish injected with 10 μM *tnnt2a*-MO2 at 60 hpf. Movie S10: the morphant shows low FI (Fig. S2K) (AVI 6979 kb)

Supplemental movie S11. Representative movies of the heart in zebrafish injected with 10 μM *tnnt2a*-MO2 at 60 hpf. Movie S11: the morphant shows intermediate FI (Fig. S2L) (AVI 6807 kb)

Supplemental movie S12. Representative movies of the heart in zebrafish injected with 10 μM *tnnt2a*-MO2 at 60 hpf. Movie S12: the morphant shows high FI (Fig. S2M) (AVI 6097 kb)
